# Seminal Plasma Microbiome Composition and Its Association with Sperm Morphology in Breeding Boars

**DOI:** 10.3390/biology15141126

**Published:** 2026-07-10

**Authors:** Notsile H. Dlamini, Serge L. Kameni, Peixin Fan, Seongbin Park, Shengfa F. Liao, Jean M. Feugang

**Affiliations:** 1 Department of Animal and Dairy Sciences, Mississippi State University, Starkville, MS 39762, USA; 2 Coastal Research and Extension Center, Mississippi State University, Biloxi, MS 39532, USA

**Keywords:** bacteria, fungi, microbiome, pig, semen, sperm quality

## Abstract

Semen quality is a key determinant of reproductive performance in breeding boars, and emerging evidence suggests the seminal microbiome may influence sperm function. However, the composition of the seminal plasma microbiome and its relationship to sperm quality remain poorly characterized. This study aimed to investigate the microbial composition of boar seminal plasma and its association with sperm quality. Semen ejaculates collected from Duroc boars were analyzed and classified as Passed (≥70% normal morphology) or Failed (<70% normal morphology). Seminal plasma was isolated by centrifugation and analyzed using 16S/ITS rRNA gene sequencing. The dominant bacterial phyla were Firmicutes, Bacteroidetes, Proteobacteria, and Actinobacteria. The most abundant genera included *Porphyromonas*, *Bacteroides*, and *Cladosporium*. Only the Shannon diversity index was significantly higher in Failed samples for the bacterial microbiome (*p* = 0.038). Furthermore, correlation analysis showed a negative association between Tenericutes and sperm concentration (r = −0.90; *p* = 0.014). Linear discriminant analysis identified microbial biomarkers associated with sperm quality, including *Rhodococcus*, *Sphingomonas*, *Lactobacillus*, *Streptococcus*, and *Empedobacter*. The increased abundance of these genera in Failed samples suggests disruption of the normal seminal microbial community. In conclusion, boar seminal plasma harbors a distinct bacterial and fungal microbiome that is associated with sperm morphology.

## Simple Summary

Semen comprises the animal’s microbiota as well as environmental contaminants. However, the interrelationship between seminal plasma microbiota and sperm quality is not well defined. This study investigated the microorganisms in boar seminal plasma and their relationship to differences in sperm quality (Passed: good quality vs. Failed: poor quality). The most common bacterial phyla were Firmicutes, Bacteroidetes, Proteobacteria, and Actinobacteria. Some genera, such as *Porphyromonas* and *Bacteroides*, were highly abundant. Samples with poorer sperm quality had higher microbial diversity, suggesting an imbalance in the microbial community. A strong negative relationship was found between the bacterial phylum Tenericutes and sperm concentration, suggesting that higher levels of these bacteria were associated with lower sperm concentration. Several other bacteria were also more prevalent in low-quality samples, indicating possible dysbiosis (an unhealthy microbial balance). In conclusion, this study shows that boar seminal plasma contains a complex microbiome that can influence sperm quality, potentially enhancing or impairing boar reproductive performance. Future research using shotgun metagenomic sequencing and larger sample sizes across various pig breeds is needed to better understand the genomic content of the microbiome and its impacts on semen quality.

## 1. Introduction

In the swine industry, semen quality is a critical determinant of boar reproductive efficiency, directly influencing the success of artificial insemination (AI) programs and, ultimately, the profitability of commercial swine production. [1,2]. High-quality semen plays a key role in boar reproductive efficiency by increasing conception and fertility rates, with farrowing rates reaching 96% and increased litter sizes [3], thereby enhancing pig production. This makes semen evaluation an essential procedure for selecting ejaculates with high fertility for use in AI [4,5]. Semen comprises distinct microbial populations, including bacteria, fungi, and opportunistic pathogens, which can adversely affect sperm quality and function [6,7]. A study by Martín et al. [8] reported that 99% of semen ejaculates contained Gram-negative bacteria, such as *Enterobacter* sp., *Escherichia coli*, *Proteus* sp., *Pseudomonas* sp., and Gram-positive bacteria, including *Bacillus* sp., *Staphylococcus* sp., and *Streptococcus* sp., associated with various effects on sperm function. While AI centers maintain strict hygiene protocols, such as the double-glove collection technique, bacterial contamination (bacteriospermia) remains a problem during semen collection and processing [9,10]. To mitigate the harmful effects of bacteria on sperm quality during storage, antibiotics are commonly added to semen extenders [11].

Bacterial contamination can stem from the skin, preputial diverticulum, fecal matter, hair, and the semen handling environment. Key sources during collection include the boar’s foreskin, indoor air, and processing equipment [9,12,13]. Some bacteria are introduced into semen as it passes through the urethra during ejaculation, with most of these microbes originating from the environment rather than from the boars themselves [14,15]. These contaminants can also be transmitted to the female reproductive tract during natural mating [16,17,18]. These bacteria in semen can negatively affect sperm quality through metabolic byproducts produced by viable bacteria and by competing for nutrients in semen extenders during storage [10,17,19,20]. Moreover, fungi also represent an important group of microorganisms that may originate from the boar’s foreskin or the surrounding indoor environment [12]. Their presence has been associated with reproductive tract infections and pregnancy losses in horses [21].Traditionally, bacterial culture-based methods, which are considered the gold standard, have been used to quantify colony-forming units (CFU/mL) and identify microbial species in boar semen [7,10,22]. The most frequently isolated genera include *Staphylococcus*, *Proteus*, and *Micrococcus* [8,23]. However, these culture-based methods are limited in their ability to detect unculturable bacteria and do not fully account for the correlations between the semen microbiome and sperm quality [24].

Advances in 16S rRNA sequencing and bioinformatic analysis now offer greater taxonomic resolution and the ability to identify a broader range of bacterial species, providing more detailed insights into the microbial composition of boar semen [25,26]. Research on the boar seminal microbiome is still in its early stages, with only a few studies exploring the relationship between the semen microbiome and sperm quality [23,26,27]. Studies by Zhang [26] and Gòdia et al. [28] identified four main phyla, including Proteobacteria, Firmicutes, Actinobacteria, and Bacteroidetes, as dominant components of the semen microbiome, with implications for semen quality and sperm fertilizing capacity. Recently, the microbiome of 17 boar semen ejaculates under tropical conditions was characterized, revealing 17 bacterial phyla, predominantly Firmicutes, Proteobacteria, Actinobacteriota, and Bacteroidota, as well as 270 genera across all samples [23].Despite growing interest in the porcine semen microbiome, research remains limited.

The relationship between the seminal microbiota and variation in sperm quality, particularly sperm morphology, in boars has not been thoroughly investigated. We hypothesize that shifts in the microbial composition and diversity of boar seminal plasma are associated with differences in semen quality, as reflected in sperm morphology. The objective of this study was to characterize the microbial composition of boar seminal plasma and to evaluate their association with sperm quality.

## 2. Materials and Methods

### 2.1. Animals

A total of 45 semen ejaculates were collected from healthy, proven fertile Duroc breeding boars (*n* = 45), routinely used in commercial artificial insemination, aged 1 to 2.5 years, with each boar contributing one ejaculate. All boars were housed in hygienic, individual pens within a closed facility at a commercial boar stud (Prestage Farms Inc., West Point, MS, USA) equipped with a ventilated cooling system. Environmental conditions in the barn were maintained at an average temperature of 20.5 ± 0.1 °C and relative humidity of 61.2 ± 2.2%. Each boar received a standard commercial diet daily and had unlimited access to water. Semen collection at the boar stud was routinely performed in a hygienic room by a trained technician using a sterile artificial vagina to manually collect semen from boars with thoroughly cleaned prepuces and surrounding areas. Immediately after collection, an aliquot (10 mL) of semen from each boar was transferred into a sterile 50 mL tube, which was donated for this research from March to April 2025.

### 2.2. Semen Processing and Evaluation

Semen aliquots were chill-transported to the laboratory at 17–19 °C within two hours and then incubated in a 37 °C water bath for 15 min. Sperm concentration was determined using Spermacue^®^ (Minitube, Tiefenbach, Germany). Subsequently, 2 µL of diluted semen was loaded onto pre-warmed, caffeine-free microscope slides (Leja^®^ Standard Count 4-chamber Slide, 20 µm, Nieuw-Vennep, The Netherlands). Sperm parameters, including total and progressive motility, normal morphology, straight-line velocity (VSL), curvilinear velocity (VCL), and average path velocity (VAP), were assessed using a computer-assisted sperm analysis system (CEROS II, IMV Technologies, Brooklyn Park, MN, USA) under a phase-contrast microscope at 37 °C on a heated stage (CX-41, Olympus, Tokyo, Japan). The system operated at 60 Hz (60 frames per second). Sperm motility was categorized based on VAP > 45 µm/s and VSL > 5 µm/s, while slow-moving cells were defined as those with VAP < 20 µm/s and VSL < 5 µm/s. Progressive sperm were defined as those with VAP > 45 µm/s and straightness > 45%, with the system set to a magnification of 1.89×. A total of 1500 sperm cells, randomly selected from five fields, were analyzed per sample to assess motility.

### 2.3. Sample Grouping

Samples were then classified according to the boar stud cut-off standard for semen quality, set at ≥70% motility and/or >70% normal morphology. Semen that did not meet these standards, usually discarded, was classified as Failed, while those that met the cut-off criteria, usually retained for artificial insemination, were classified as Passed. All samples showed comparable total motility (>70%). Therefore, based on normal morphology data, samples with the lowest values in the Failed group (≤mean − 2SD; *n* = 10) and the highest values in the Passed group (≥ mean + 2SD; *n* = 10) were used for microbial analysis (Table 1).

### 2.4. Bacteria Identification Using Bacterial Culture in Boar Seminal Plasma

**Figure 1:**
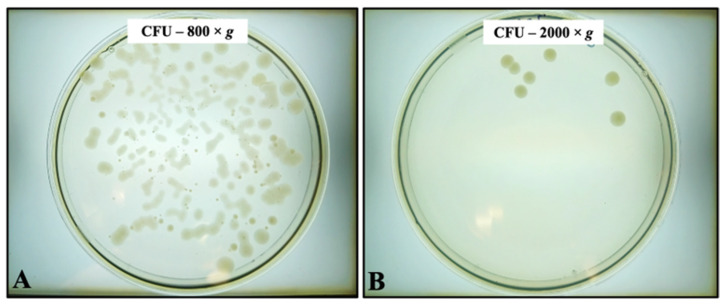
Bacterial counts (CFU/mL) on agar plates inoculated with boar seminal plasma isolated by centrifugation at (**A**) 800× *g* and (**B**) 2000× *g* for 20 min at 4 °C. Images are representative of five tests using individual boar seminal plasma aliquots (*n* = 5 for each centrifugation speed).

To test for the presence of live bacteria in seminal plasma, 2 mL of freshly collected boar semen (*n* = 5) was centrifuged at 350× *g*, 800× *g*, or 2000× *g* for 20 min at 4 °C to ensure suitable conditions for microbiome analysis. The resulting supernatants, corresponding to seminal plasma (SP), were carefully collected into sterile microcentrifuge tubes. Aliquots of SP were examined under a microscope (Eppendorf Centrifuge 5810R, refrigerated; Thermo Fisher Scientific, Waltham, MA, USA), and only SP derived at 350× *g* showed sperm carryover; therefore, SP derived at 800× *g* and 2000× *g*, with no sperm carryover, were used for bacterial culture. Aliquots (1 mL) of each sample were centrifuged at 15,000× *g* for 10 min at 4 °C to pellet bacterial cells. Supernatants were discarded, and each pellet was resuspended in 200 µL of sterile phosphate-buffered saline (PBS; Thermo Fisher Scientific, Waltham, MA, USA) by gentle vortexing and/or repeated pipetting to ensure thorough homogenization. Aliquots (100 µL) of the bacterial suspension were plated onto Tryptic Soy agar plates (Thermo Fisher Scientific, Waltham, MA, USA) and incubated for 24 h at 37 °C under 5% CO_2_. After incubation, bacterial colonies on plates were counted to determine concentration, calculated by multiplying the number of colonies by the dilution factor and dividing by the volume plated, then reported as colony-forming units per milliliter (CFU/mL). The higher abundance of bacterial colonies on agar plates inoculated with SP derived from 800× *g* centrifugation confirmed the presence of aerobic bacteria in SP from raw semen (Figure 1) and supported the selection of these SP samples for DNA extraction and 16S and ITS amplicon sequencing.

### 2.5. DNA Extraction

DNA extraction was carried out using the PureLink Microbiome DNA Purification Kit, following the manufacturer’s instructions (Thermo Fisher Scientific, Carlsbad, CA, USA). Briefly, 1 mL of seminal plasma was centrifuged at 14,000× *g* for 10 min to pellet microbial cells. The resulting pellet was lysed and incubated at 65 °C for 10 min, then homogenized by bead beating at maximum speed in a vortex mixer (Thermo Fisher Scientific, Carlsbad, CA, USA) for 10 min. Samples were then centrifuged again at 14,000× *g* for 5 min, and the supernatant was transferred to a clean tube for DNA binding. DNA was purified using a series of wash steps by centrifugation at 14,000× *g* for 1 min, followed by elution for downstream applications. The NanoDrop™ One Spectrophotometer (Thermo Scientific, Waltham, MA, USA) was used to assess the quality and concentration of extracted DNA, which was then stored at −80 °C until further processing.

### 2.6. 16S/ITS Amplicon Sequencing

Bacterial 16S rRNA gene-targeted sequencing was performed using the Quick-16S^TM^ Plus NGS Library Prep Kit (Zymo Research, Irvine, CA, USA), targeting the V3–V4 region of the 16S rRNA gene. The Quick-16S^TM^ Primer Set V3–V4 (Zymo Research, Irvine, CA, USA), custom-designed by Zymo Research, was used. DNA samples for ITS amplicon sequencing were prepared using the Quick-ITS^TM^ Plus NGS Library Prep Kit (Zymo Research, Irvine, CA, USA), with custom ITS2 primers (Microbiome Sequencing Services ITS2 Primer Set (Zymo Research, Irvine, CA, USA)). The sequencing library was prepared using an innovative library preparation process in which PCR was performed on real-time PCR instruments (Thermo Fisher Scientific, Waltham, MA, USA) to control the number of amplification cycles and minimize chimera formation. Final PCR products were quantified by qPCR fluorescence and then pooled at equal molar concentrations. The final pooled library was purified using the Select-a-Size DNA Clean & Concentrator^TM^ (Zymo Research, Irvine, CA, USA) and then quantified using both TapeStation^®^ (Agilent Technologies, Santa Clara, CA, USA) and Invitrogen Qubit 1^®^ dsDNA High-Sensitivity Assay Kits^®^ (Thermo Fisher Scientific, Waltham, MA, USA). For quality control, the ZymoBIOMICS^®^ Microbial Community DNA Standard (Zymo Research, Irvine, CA, USA) was included as a positive control for each targeted library preparation. Negative controls (blank extraction and blank library preparation controls) were used to monitor potential contamination during the wet-lab process. Sequencing was carried out on an Illumina^®^ Nextseq^TM^ (Illumina, San Diego, CA, USA) using a P1 reagent kit (600 cycles), with a 30% PhiX spike-in.

### 2.7. Bioinformatic Analysis

Unique amplicon sequence variants (ASVs) were inferred from raw reads using the DADA2 pipeline, v.1.26.0 [28], which also removed potential sequencing errors and chimeric sequences. Taxonomic assignment was carried out using Uclust from QIIME v.1.9.1 [29] with the Zymo Research 16S and ITS databases. Composition visualization and alpha diversity indices (Chao1, Shannon, Observed, and Simpson) were calculated from ASV counts using QIIME v.1.9.1 to assess species richness and evenness across samples. The principal coordinate analysis (PCoA) based on Bray–Curtis dissimilarity using unique ASVs was performed with QIIME v.1.9.1 to assess beta diversity. Differentially abundant taxa across groups were identified using linear discriminant analysis effect size (LEfSe) and visualized as cladogram plots [30], with default parameters.

### 2.8. Absolute Abundance Quantification

Quantitative real-time PCR (qPCR) was performed using a standard curve approach to enable absolute quantification of target gene copies. The standard curve was constructed using plasmid DNA carrying single-copy targets for the bacterial 16S rRNA gene and the fungal ITS2 region. The resulting standard curve equation was used to calculate the number of gene copies per reaction for each sample. To determine gene copy concentration in the original DNA extracts, the calculated gene copy number per reaction was normalized to the PCR input volume (2 μL), yielding gene copies per microliter of DNA sample. Genome copy number/μL was then estimated by dividing gene copy number by the assumed number of target gene copies per genome (4 copies for 16S rRNA genes and 200 copies for ITS regions). Total DNA concentration (g/μL) was subsequently estimated from genome copy number, assuming genome sizes of 4.64 × 10^6^ base pairs for bacterial samples (based on Escherichia coli) and 1.20 × 10^7^ base pairs for fungal samples (based on Saccharomyces cerevisiae). Calculations incorporated the average molecular weight of a DNA base pair (660 g mol^−1^ bp^−1^) and Avogadro’s number (6.022 × 10^23^ mol^−1^), according to the following equation: Calculated DNA concentration = (genome copies per μL) × (genome size in bp) × (660 g mol^−1^ bp^−1^) ÷ (6.022 × 10^23^ mol^−1^).

### 2.9. Statistical Analysis

All sperm data were analyzed using R statistical software version 4.5.2 (Boston, MA, USA) and MicrobiomeAnalyst (https://www.microbiomeanalyst.ca/, version 2.0, accessed 8 September 2025). The Shapiro–Wilk test was used to assess the normality of the sperm motion data. Semen characteristics between Passed and Failed semen samples were analyzed using the Wilcoxon rank-sum test. Alpha-diversity indices (Chao1, Shannon) were analyzed using the Wilcoxon rank-sum test and used for pairwise comparisons between groups on MicrobiomeAnalyst (https://www.microbiomeanalyst.ca/, version 2.0, accessed 12 September 2025). Beta diversity was analyzed using permutational multivariate analysis of variance (PERMANOVA). *p*-values (0.05) were adjusted using the Benjamini–Hochberg false discovery rate (FDR) procedure where necessary. The Kruskal–Wallis rank-sum test, coupled with linear discriminant analysis effect size (LEfSe), was applied to identify bacterial biomarkers that differed significantly between the two groups, given their minimum sample sizes of 10 each. Spearman’s correlation with Bonferroni Correction of the unadjusted Spearman *p*-values was used to analyze associations between sperm quality parameters and the most abundant bacterial/fungal phyla and alpha diversity indices. A significant difference was set at *p*-value < 0.05.

## 3. Results

### 3.1. Semen Quality Analysis

On average, total motility, progressive motility, normal morphology, concentration, and proximal droplet were significantly higher in the Passed semen group compared to the Failed group (*p* < 0.05; Table 1).

### 3.2. Absolute Abundance

**Figure 2:**
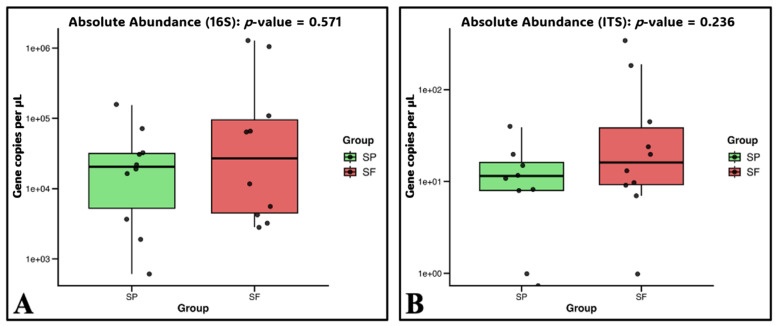
Absolute abundance of gene copies in bacterial 16S rRNA (**A**) and fungal ITS region (**B**) in Passed (SP) and Failed (SF) seminal plasma groups.

To account for microbial composition based on sequencing-derived data, gene copy numbers for bacterial 16S rRNA and fungal ITS targets were quantified and used to estimate total microbial DNA per sample. On average, Passed seminal plasma samples had 35,229.7 ± 14,786.0 gene copies/µL for 16S rRNA, whereas Failed samples had 261,284.9 ± 153,363.7 gene copies/µL. In contrast, ITS gene copies for fungi were 12.7 ± 3.9 in Passed samples and 66.2 ± 35.5 in Failed samples. No significant differences were observed in bacterial (*p* = 0.571; Figure 2A) or fungal (*p* = 0.236; Figure 2B) DNA between seminal plasma groups. Neither the blank extraction (negative control) nor the blank library preparation (negative control) produced any bacterial or fungal genome copies (Supplementary Material Table S1).

### 3.3. Taxonomic Profiles of Boar Seminal Plasma Microbial Communities

A total of 25 bacterial phyla, 46 classes, 78 orders, 140 families, 429 genera, and 2013 species were identified through 16S amplicon sequencing, while ITS amplicon sequencing detected 4 fungal phyla, 11 classes, 17 orders, 25 families, 28 genera, and 35 species (Table 2).

**Figure 3:**
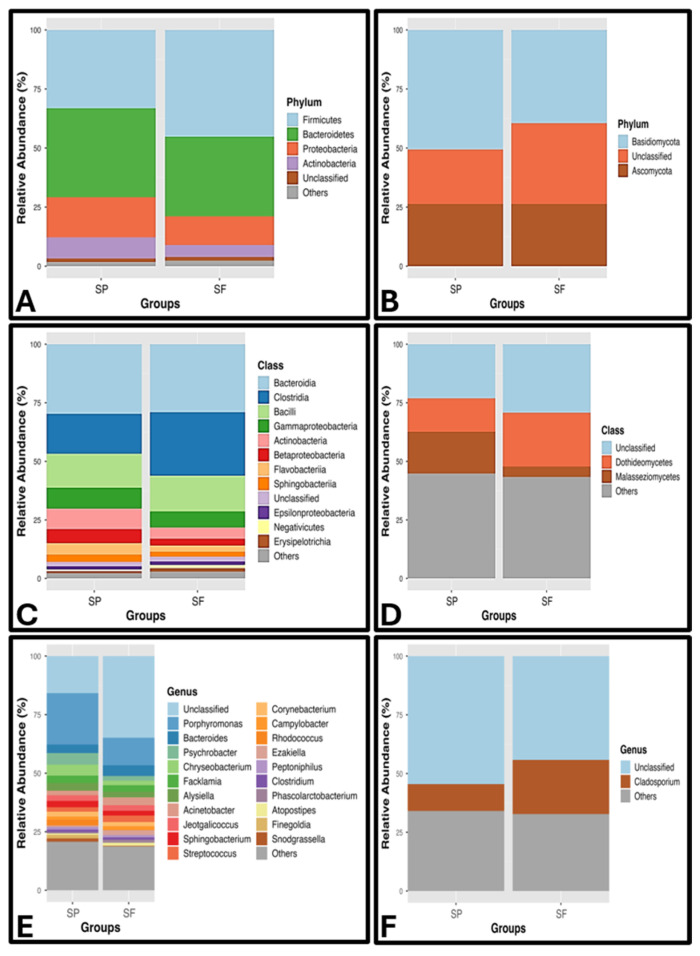
Relative abundance of the most prevalent (≥50%) bacterial and fungal phyla (**A**,**B**), class (**C**,**D**), and genera (**E**,**F**) between Passed (SP) and Failed (SF) seminal plasma groups.

The most abundant microbial taxa were selected based on ≥50% prevalence and >1% relative abundance. The most predominant bacterial phyla were Actinobacteria, Bacteroidetes, Firmicutes, and Proteobacteria. Firmicutes exhibited the highest relative abundance, with 33.2% vs. 45.2% between SP and SF groups, respectively (Figure 3A). This was followed by Bacteroidetes (37.6% vs. 33.7%), Proteobacteria (17% vs. 12.1%), and Actinobacteria (8.9% vs. 5.1%). Among the fungal phyla, Basidiomycota was the most abundant (50.6% vs. 39.5%), followed by Ascomycota (26.3% vs. 26.3%) and unclassified (23.1% vs. 34.2%) (Figure 3B). No significant differences in relative abundance of these microbial phyla were observed between the semen quality groups (*p* > 0.05).

At the bacterial class level, Bacteroidia (29.7% vs. 29.0%) and Clostridia (17% vs. 27.2%) were the most abundant (Figure 3C). Among fungal classes, unclassified (23.1% vs. 33.0%) and Dothideomycetes (14.2% vs. 23.0%) were most abundant in the SP and SF groups, respectively (Figure 3D). Notably, at the bacterial genus level, unclassified (15.8% vs. 34.9%) and Porphyromonas (21.9% vs. 11.8%) were the most abundant (Figure 3E). For fungal genera, unclassified was most prevalent (54.5% vs. 44.2%), followed by Cladosporium (11.4% vs. 25.0%), which was more common in the SF group (Figure 3F). Similarly, no significant differences (*p* > 0.05) were observed at the class and genus level for either the bacterial or fungal microbiome.

### 3.4. Microbial Diversity of Boar Seminal Plasma

**Figure 4:**
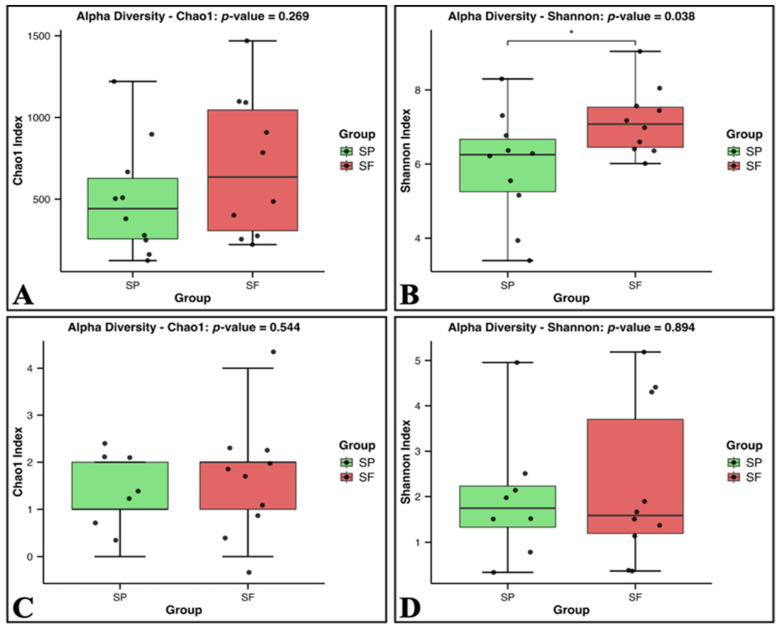
Alpha diversity measures of bacterial (upper panel) and fungal (bottom panel) communities between Passed (SP) and Failed (SF) groups. Chao1 index (**A**,**C**) and Shannon’s index (**B**,**D**) values between Passed (SP) and Failed (SF) groups. Asterisk (*) indicates significant differences (*p* < 0.05).

Regarding alpha diversity, there were no significant differences in bacterial richness between the Passed and Failed semen quality groups, as indicated by Chao1 index (*p* = 0.269; Figure 4A). However, bacterial richness and evenness measured by the Shannon index were significantly higher (*p* = 0.038) in Failed samples (Figure 4B). For the fungal community, no significant differences were observed in the Chao1 index (*p* = 0.544; Figure 4C) or Shannon index (*p* = 0.894; Figure 4D).

**Figure 5:**
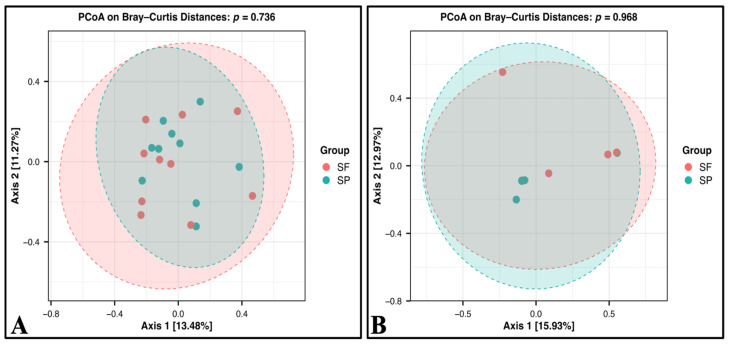
Beta diversity of bacteria (**A**) and fungal (**B**) communities visualized using Principal Coordinates Analysis (PCoA) based on Bray–Curtis distances between Passed (SP) and Failed (SF) seminal plasma groups.

Beta diversity analysis, based on Bray–Curtis dissimilarity, also showed no significant differences in diversity of bacteria (*p* = 0.736; Figure 5A) or fungal (*p* = 0.968; Figure 5B) communities between seminal plasma groups.

### 3.5. Identification of Microbial Biomarkers Associated with Semen Quality

**Figure 6:**
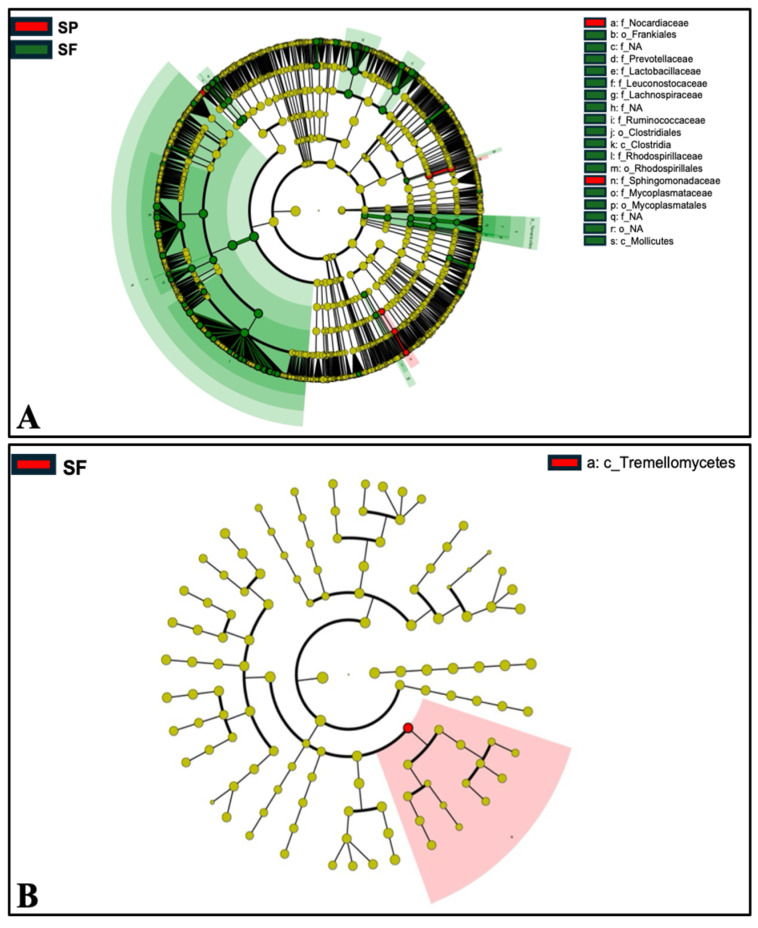
Linear discriminant effect size (LEfSe) cladogram depicting bacteria (**A**) and fungal (**B**) biomarkers between Passed (SP) and Failed (SF) boar seminal plasma groups.

**Figure 7:**
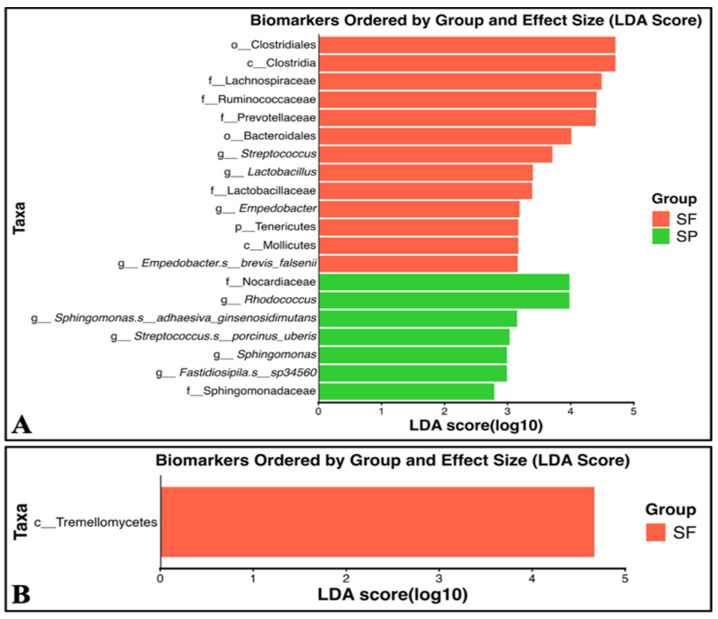
Linear discriminant analysis (LDA) score plot showing (**A**) bacterial and (**B**) fungal biomarkers between Passed (SP) and Failed (SF) boar seminal plasma groups.

Bacterial and fungal biomarkers were identified in the boar seminal plasma microbiome using linear discriminant effect size (LEfSe) and visualized through a cladogram (Figure 6) and an LDA score plot (Figure 7). Of the 97 identified bacterial biomarkers (Supplementary Material Table S2), 20 were deemed most significant based on an LDA score threshold of > 2.8, with 7 linked to the SP group and 13 to the SF group. In the SP group, the families Sphingomonadaceae and Nocardioidaceae, as well as the genera *Rhodococcus* and *Sphingomonas*, were significantly enriched (*p* < 0.05). In contrast, the SF group showed enrichment (*p* < 0.05) in the phylum Tenericutes, the classes Clostridia and Mollicutes, the orders Clostridiales and Bacteroidales, the families Lachnospiraceae, Ruminococcaceae, Prevotellaceae, and Lactobacillaceae, and the genera *Streptococcus*, *Lactobacillus*, and *Empedobacter* (Figure 7A). Among fungi, only the class Tremellomycetes was significantly enriched in the SF group (*p* < 0.05; Figure 7B).

### 3.6. Correlation Analysis Between Major Phyla and Alpha Diversity Metrics

**Figure 8:**
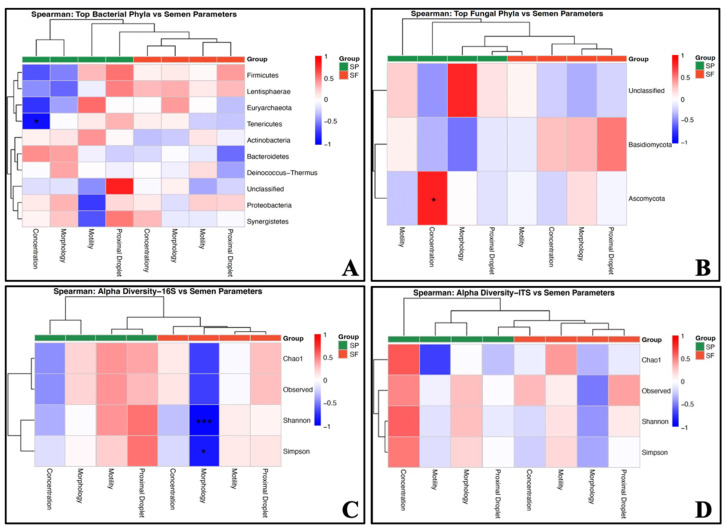
Correlation heatmap of semen quality parameters with the topmost abundant bacteria (**A**), fungal phyla (**B**), alpha diversity metrics of bacterial (**C**) and fungal (**D**) microbiome between Passed (SP) and Failed (SF) boar seminal plasma samples. Asterisks indicate significant differences (*: *p* < 0.05; ***: *p* < 0.001).

Spearman correlation analysis was conducted within each group to examine relationships between key semen parameters (motility, normal morphology, concentration, and proximal droplet) and the most prevalent bacterial and fungal phyla, as well as alpha-diversity metrics of the bacterial and fungal microbiomes. A negative correlation was observed only between Tenericutes and sperm concentration (r = −0.90; *p* = 0.014) within the bacterial microbiome (Figure 8A). In the fungal microbiome, Ascomycota was positively correlated with sperm concentration (r = 0.80; *p* = 0.027; Figure 8B). Additionally, significant negative correlations were observed between normal morphology and bacterial microbiome alpha-diversity metrics, including Shannon (r = −0.95; *p* < 0.001) and Simpson (r = −0.86; *p* = 0.012; Figure 8C). In contrast, no significant correlations were found between semen parameters and alpha diversity metrics of the fungal microbiome (Figure 8D). Scatter plots for significant correlations are provided in Figure S1.

## 4. Discussion

Bacteria in boar semen is known to negatively affect sperm quality and may also pose an infection risk during artificial insemination (AI) of sows [29]. These microorganisms can cause both direct and indirect harmful effects on sperm cells, affecting sperm movement, survival, and fertilizing ability [10,30]. In this study, 16S rRNA and ITS sequencing were applied to characterize the seminal plasma microbiome and evaluate its potential impact on semen quality in boars. Although previous research has shown links between the seminal microbiota and sperm quality parameters in boars [10,23,31], few studies have specifically investigated the microbial communities in seminal plasma [31], especially semen of varying quality. Our earlier work demonstrated that semen categorized as Failed or Passed according to sperm motility and/or normal morphology exhibits distinct metabolomic profiles [32,33]. These profiles may include bacterial-derived metabolites whose role in semen quality variation remains unknown.

In the present study, restricted to sperm morphology as the main cut-off criterion of Passed and Failed samples, we found that the predominant bacterial phyla across Passed and Failed groups were Firmicutes (33.2% vs. 45.2%), Bacteroidetes (37.6% vs. 33.7%), Actinobacteria (8.9% vs. 5.1%), and Proteobacteria (17% vs. 12.1%), indicating their potential roles in supporting sperm function. This finding is consistent with previous reports in boars and humans [26,28,34]. In boar semen, four bacterial phyla were identified as dominant, including Firmicutes (49.5%), Proteobacteria (22.6%), Actinobacteria (12.5%), and Bacteroidetes (12.4%) in both high- and low-quality boar semen samples [23]. At the class level, Bacteroidia (29.7% vs. 29%), Clostridia (17% vs. 27.2%), and Bacilli (14.5% vs. 15.2%) were the most prevalent, with *Porphyromonas* (21.9% vs. 11.8%), *Bacteroides* (3.6% vs. 4.6%), and *Acinetobacter* (1.9% vs. 3.4%) being the dominant bacterial genera. These findings agree with those of [11], who reported similar dominant taxa in the boar’s seminal microbiome. Within the phylum Firmicutes, the class Clostridia (27.2%) was more abundant in Failed seminal plasma samples. Clostridia species are Gram-positive bacteria and include certain opportunistic pathogens; their increase may signal dysbiosis or a shift away from a healthy microbial community in boar semen. The presence of Clostridia in semen can naturally occur, but high levels due to contamination during collection and processing can be harmful, leading to decreased sperm quality (e.g., motility, viability, membrane integrity, increased agglutination) [27,35,36,37,38]. This mechanism could be triggered by endotoxins secreted by specific *Clostridium* strains, which cause physiological changes in sperm cells, ultimately reducing sperm quality [39].

Within the phylum Bacteroidetes, the class Bacteroidia has been shown to be negatively associated with sperm concentration and total sperm count, suggesting that specific genera within Bacteroidia may be pathogenic [40]. Similarly, Bacilli, a class of Gram-positive bacteria within the Firmicutes phylum, were more abundant in Failed samples. Semen containing this bacterium has been associated with reproductive failure following insemination [41]. Their presence in the semen of healthy, fertile males warrants further studies to better understand their roles in reproductive health [34].

Additionally, the anaerobic *Porphyromonas* genus, a common bacterium found in the semen of both fertile and infertile men, was observed at higher levels in Passed boar seminal plasma. This contrasts with its reported association with poor semen quality, including increased DNA fragmentation, impaired sperm motility and morphology, and reduced sperm concentration due to reactive oxygen species production [42]. This discrepancy could be due to species specificity (e.g., pig versus human) or the *Porphyromonas* species. While a recent report indicated a positive association between *Porphyromonas assaccharolytica* and sperm concentration in men [34], the abundance and dynamics of bacterial communities affecting sperm quality vary within the urogenital tract during puberty (pre-pubertal vs. post-pubertal), between boar studs (e.g., Proteobacteria, Firmicutes, Bacteriodetes, Actinobacteria), and during post-collection storage (e.g., *Prevotella*, *Ruminococcus*, and *Bacteroides*) in swine [27,43].

Regarding the fungal community, the phyla Ascomycota (26.3%) and Basidiomycota (50.6% vs. 39.5%) were predominant, with Basidiomycota showing the highest relative abundance in both semen quality groups. The most common fungal class was Dothideomycetes in Failed samples (23.0%) and Maiasseziomycetes in Passed samples (17.9%), while the only observed predominant genus was *Cladosporium* in Failed samples (25.0%). Currently, there are few studies on fungal contamination in boar semen, underscoring the novelty and significance of the present investigation. A recent study found in the blood plasma of men with prostate cancer reduced abundance of the phylum Dothideomycetes and the genus *Cladosporium* when compared with healthy men [44]. The presence of these fungi in semen might originate from the urinary tract [45] as well as from environmental sources such as indoor air or the boar’s preputial mucosa [12]. *Cladosporium* is the most common fungal species found [46], and is often highly abundant in outdoor air. Its abundance in boar seminal plasma could be linked to environmental contamination and to the internal male reproductive tract.

The microbial diversity and richness (alpha diversity) of boar seminal plasma were examined to compare semen quality across groups. Only the Shannon index was significantly higher in the Failed samples, indicating greater species richness and a more even microbial distribution in lower-quality semen. While this pattern may suggest that a more diverse seminal plasma microbiome is associated with poorer sperm morphology outcomes, this interpretation warrants caution. Increased microbial diversity is not universally indicative of a detrimental or dysbiotic state; in some biological contexts, higher diversity reflects a more resilient and stable microbial ecosystem [47]. In contrast, beta diversity (differences in microbial community composition between samples) showed that Bray–Curtis dissimilarity was not significant for either the bacterial or the fungal datasets.

This finding suggests that the morphology-associated microbial signatures identified through differential abundance and LEfSe analyses reflect shifts in the relative abundance of specific taxa rather than in whole-community restructuring. These results align with those of [25] and [23], who reported no significant differences in beta diversity or overall compositional shifts between boar semen samples with different semen quality. Such discrepancies may be due to environmental or management factors, including variations in housing, hygiene, or semen collection environments. Indeed, Mulder et al. [48] demonstrated clear associations between the intestinal microbiota of pigs and their surrounding environments, supporting the influence of external factors on reproductive tract microbiota.

Based on the linear discriminant analysis effect size (LEfSe), several bacterial taxa could serve as indicators for distinguishing Passed from Failed semen samples. Notably, the families Nocardiaceae and Sphingomonadaceae, as well as the genera *Rhodococcus*, and *Sphingomonas*, were more abundant in the Passed semen group. The abundance of the bacterial family Sphingomonadaceae, which is part of the Gram-negative phylum *Proteobacteria*, has been linked to semen quality (e.g., normal viscosity, sperm morphology, and DNA protamination/compaction), although the exact roles may vary depending on the specific species within the family or the overall microbial balance in the semen [49,50]. The *Sphingomonas* genus is commonly present in healthy human semen and is positively associated with various sperm motility parameters [49]. Its higher abundance has been identified as a potential microbial indicator for semen quality in goats [51].

In contrast, the Failed seminal plasma samples showed enrichment of the phylum Tenericutes and the genera *Streptococcus*, *Lactobacillus*, and *Empedobacter*. Our results support previous reports indicating a negative correlation between increased Tenericutes abundance and sperm motility and acrosome integrity [52]. While the enrichment of Tenericutes in Failed samples is an interesting observation, we cannot conclude from the present data that this taxon represents an indicator for male infertility or subfertility. This warrants further research to clarify its role in sperm quality. The role of *Streptococcus* in semen quality appears to be context-dependent. While several studies have linked its presence to reduced sperm motility and morphology [22,53,54], other studies have reported a positive association. For example, a comparison of semen microbiota between two boar studs found a higher relative abundance of *Streptococcus* in the stud with superior sperm quality parameters [27]. The contrasting findings observed here may reflect differences in *Streptococcus* species or strain composition across studies, as 16S rRNA sequencing targeting the V3–V4 hypervariable regions does not provide species- or strain-level resolution. This limitation precludes definitive conclusions regarding the specific *Streptococcus* taxa present in Failed versus Passed samples, and whole-genome shotgun sequencing would be required to resolve this ambiguity [22,27].

The present findings for *Lactobacillus* differ from studies that report a positive association with sperm quality and reproductive performance in boars [27,55,56]. While *Lactobacillus* is generally regarded as a beneficial constituent of the normo-spermic microbiome, its role appears to be species- and context-dependent. A recent study identified higher abundance of *Lactobacillus iners* in the semen of men with abnormal sperm quality [55], suggesting that not all *Lactobacillus* species confer protective effects. It is possible that the *Lactobacillus* taxa enriched in our Failed samples represent species with neutral or detrimental effects on sperm quality, rather than the probiotic species (e.g., *L. acidophilus*, *L. salivarius*, *L. reuteri*) associated with improved sperm parameters in other species [52]. However, because our 16S rRNA sequencing data do not provide species-level resolution for *Lactobacillus*, this interpretation remains speculative and warrants investigation in future studies using higher-resolution sequencing approaches. At present, little is known about the genus *Empedobacter* in livestock semen aside from its unique detection in turkey semen [57].

Within the fungal community, only the class Tremellomycetes was significantly enriched in Failed samples. Tremellomycetes, a class within the subdivision Agaricomycotina, is one of the three main lineages of the Basidiomycota (fungi that produce spores on basidia). It includes both dimorphic yeasts and filamentous species capable of forming hyphae or complex fruiting bodies [58,59]. Their increased abundance may suggest fungal contamination during semen collection, handling, or storage, which could compromise sperm quality (e.g., motility and viability) and cause DNA fragmentation. Certain members of this class, including *Trichosporon* and *Cryptococcus*, are recognized opportunistic pathogens [60] capable of producing enzymes and bioactive metabolites that may impair sperm physiology [61]; however, whether the Tremellomycetes detected in the present study belong to pathogenic genera was not determined, and direct evidence of a causal relationship with sperm morphology outcomes is lacking. Moreover, *Trichosporon* spp. have been reported as the dominant fungi in the genital tract (vagina, foreskin) and in the semen of healthy giant pandas [62], highlighting the presence of this genus across diverse mammalian reproductive microbiomes. Whether Tremellomycetes, including *Trichosporon*, play a pathological or commensal role in boar seminal plasma remains unknown.

## 5. Conclusions

This study presents an initial microbiome analysis of raw, antibiotic-free boar semen, demonstrating that boar seminal plasma harbors a diverse and intricate mixture of bacterial and fungal communities associated with sperm morphology in breeding boars. Notable differences in the distribution of specific taxa were found between Failed semen, such as Firmicutes, Clostridia, *Bacteroides*, Dothideomycetes, and *Cladosporium*, and Passed semen, which included Proteobacteria, *Porphyromonas*, and Malasseziomycetes. The research also points to potential biomarkers for semen quality: *Streptococcus*, *Lactobacillus*, Clostridia, and Tremellomycetes for Failed semen; *Sphingomonas* and *Rhodococcus* for Passed semen. These findings suggest a potential relationship between the seminal microbiome and semen quality traits; however, further studies are required to determine the functional and causal significance of these microbial associations. Despite a limited sample size and the use of a single commercial breed, this work provides important insights into the boar seminal microbiome and suggests new strategies, including shotgun metagenomic sequencing, larger cohorts, and multiple pig breeds, to improve the reproductive success of semen with poor quality through microbiome-based interventions.

## Figures and Tables

**Table 1:** Sperm characteristics between the Passed and Failed semen groups.

Parameters	Passed (*n* = 10)	Failed (*n* = 10)	*p*-Value
Total motility (%)	87.5 ± 2.1 ^a^	77.1 ± 4.0 ^b^	0.038
Progressive motility (%)	72.4 ± 3.4 ^a^	59.3 ± 4.4 ^b^	0.002
Normal morphology (%)	84.8 ± 1.6 ^a^	58.2 ± 2.4 ^b^	<0.001
Concentration (×10^6^ sperm/mL)	31.8 ± 2.3 ^a^	21.1 ± 1.9 ^b^	0.006
Average path velocity (VAP; μm/s)	50.5 ± 2.0	46.6 ± 5.8	0.791
Straight-line velocity (VSL; μm/s)	32.4 ± 2.7	33.8 ± 4.4	0.473
Curvilinear velocity (VCL; μm/s)	106.9 ± 6.7	98.7 ± 12.0	0.910
Bent tail (%)	7.1 ± 0.4	9.1 ± 1.0	0.306
Coiled tail (%)	0.6 ± 0.1	1.0 ± 0.1	0.085
Distal droplet (%)	10.0 ± 1.1	18.8 ± 4.6	0.064
Proximal droplet (%)	3.9 ± 0.4 ^a^	7.7 ± 2.2 ^b^	0.031
Linearity (%)	31.8 ± 1.6	32.7 ± 4.0	0.427
Straightness (%)	64.9 ± 2.6	66.0 ± 7.6	0.104
Amplitude head (ALH; μm)	6.1 ± 0.2	5.1 ± 0.6	0.089
Beat cross frequency (BCF; Hz)	27.7 ± 1.0	29.6 ± 3.7	0.121

^a,b^ Different superscripts in each parameter indicate significant differences (*p* < 0.05). Semen classification was based on normal morphology: Passed (≥70%) and Failed (<70%). Data are represented by mean ± SEM.

**Table 2:** Distribution of microbial taxonomy identified through 16S and ITS amplicon sequencing.

Taxonomy Level	Sequencing Type (*n*)	
	16S	ITS
Phylum	25	4
Class	46	11
Order	78	17
Family	140	25
Genus	429	28
Species	2013	35

## Data Availability

The original contributions presented in this study are included in the article/Supplementary Materials. Further inquiries can be directed to the corresponding author.

## Supplementary Materials

The following supporting information can be downloaded at: https://www.mdpi.com/article/10.3390/biology15141126/s1, Table S1: ZymoBIOMICS^®^ microbial community DNA standards and negative controls; Table S2: Bacterial biomarkers identified using linear discriminant effect size (LEfSe) between Passed (SP) and Failed (SF) boar seminal plasma groups; Figure S1: Spearman correlation scatter plots of all significant correlations and confidence intervals.

## Abbreviations

The following abbreviations are used in this manuscript:

AI	Artificial insemination
ASVs	Amplicon sequence variants
CASA	Computer assisted sperm analysis
CFU	Colony forming units
DNA	Deoxyribonucleic acid
dsDNA	Double-stranded deoxyribonucleic acid
LDA	Linear discriminant analysis
LEfSe	Linear discriminant analysis effect size
NGS	Next generation sequencing
PBS	Phosphate-buffered saline
PCoA	Principal coordinates analysis
PCR	Polymerase chain reaction
PERMANOVA	Permutational multivariate analysis of variance
qPCR	Quantitative polymerase chain reaction
ROS	Reactive oxygen species
rRNA	Ribosomal ribonucleic acid
SD	Standard deviation
SEM	Standard error of mean
SF	Seminal plasma failed
SP	Seminal plasma
SP	Seminal plasma passed
VAP	Average path velocity
VCL	Curvilinear velocity
VSL	Straight-line velocity
