# Supplementary material for: Seminal Plasma Microbiome Composition and Its Association with Sperm Morphology in Breeding Boars
Source: Biology (Basel). 2026 Jul 10;15(14):1126. doi: 10.3390/biology15141126 (PMC13403968; doi:10.3390/biology15141126)
Supplement: Supplementary file 1 [file biology-15-01126-s001.zip › biology-4405582-Figure S1.pdf]

Supplementary Material

Figure S1. Spearman correlation scatter plots of all significant correlations and confidence intervals.

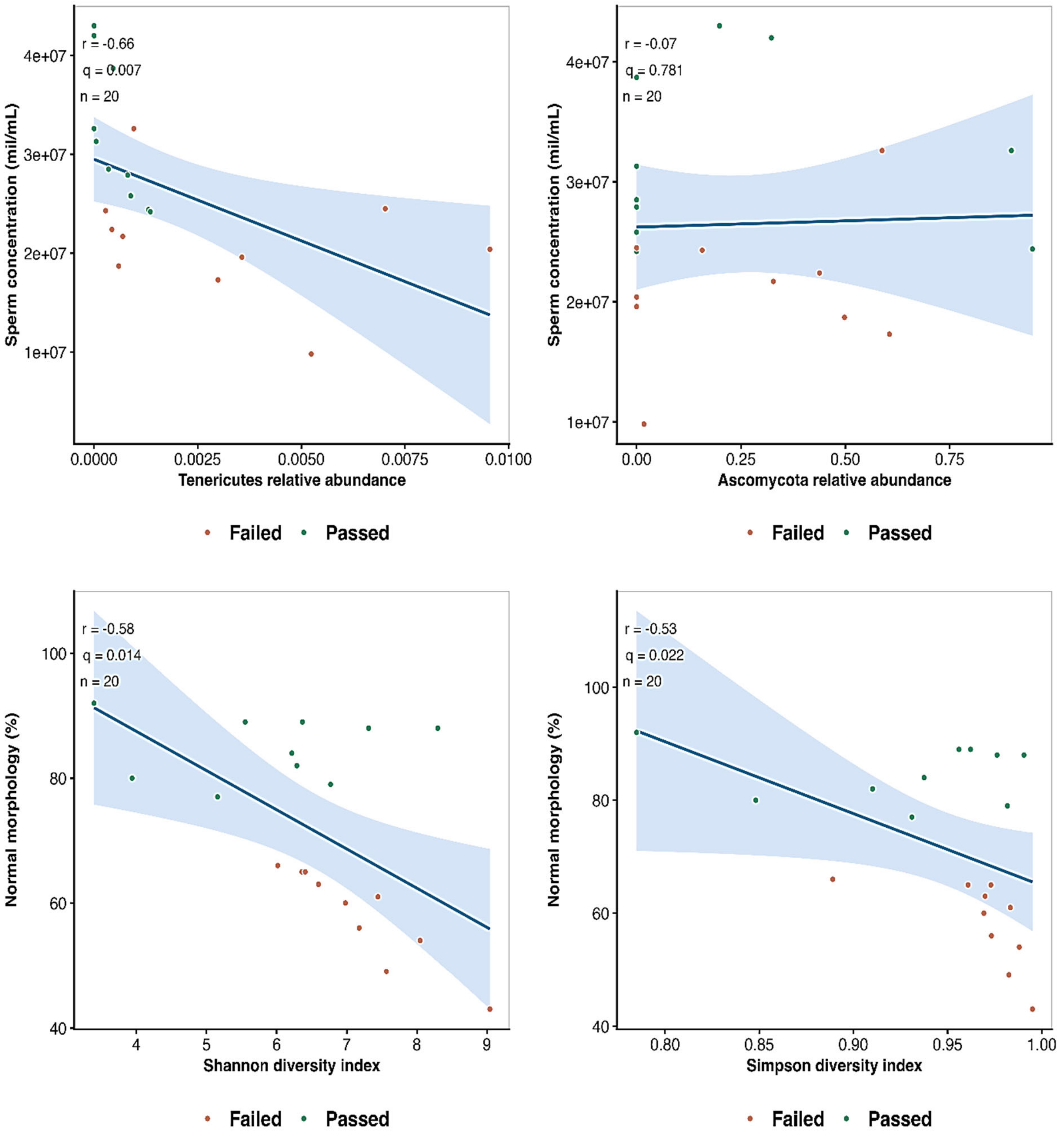

Points coloured by group.
Shaded band = 95% CI. q-values BH-adjusted.
