# Supplementary material for: Seminal Plasma Microbiome Composition and Its Association with Sperm Morphology in Breeding Boars
Source: Biology (Basel). 2026 Jul 10;15(14):1126. doi: 10.3390/biology15141126 (PMC13403968; doi:10.3390/biology15141126)
Supplement: Supplementary file 1 [file biology-15-01126-s001.zip › biology-4405582-Table S1.pdf]

Supplementary Material

Table S1. ZymoBIOMICS® microbial community DNA standards and negative controls

**16S Amplicon Sequencing**					
**sample_id**	**customer_label**	**Ct**	**gene_copies_per_μL**	**genome_copies_per__μL ***	**DNA_ng_per__μL***
Plate1P_V3V4	DnaStandard1	13.7	215771085	53942771	274.3180703
Plate1N_V3V4	LibNegative1	37.68	10	3	0.0000153
**ITS Amplicon Sequencing**					
**sample_id**	**customer_label**	**Ct**	**gene_copies_per__μL**	**genome_copies_per__μL ***	**DNA_ng_per__μL ***
Plate1P_ITS2	DnaStandard1	16.72	8479791	42399	0.5576222
Plate1N_ITS2	LibNegative1	>45	0	0	0

*Genome copies/μL and DNA ng/μL are calculated from the gene copies/μL measured in the sample.
