# Supplementary material for: Seminal Plasma Microbiome Composition and Its Association with Sperm Morphology in Breeding Boars
Source: Biology (Basel). 2026 Jul 10;15(14):1126. doi: 10.3390/biology15141126 (PMC13403968; doi:10.3390/biology15141126)
Supplement: Supplementary file 1 [file biology-15-01126-s001.zip › biology-4405582-Table S2.pdf]

Supplementary Material

Table S2. Bacterial biomarkers identified using linear discriminant effect size (LEfSe) between Passed (SP) and Failed (SF) boar seminal plasma groups.

**Taxonomic Classification**	**Group**	**LDA score**	**Log-LDA score**	***P*-value**
k__Bacteria.p__Firmicutes.c__Bacilli.o__Lactobacillales.f__Leuconostocaceae.g__Weissella	SF	3.032	2.835	0.002
k__Bacteria.p__Proteobacteria.c__Alphaproteobacteria.o__Rhodospirillales	SF	2.983	2.706	0.002
k__Bacteria.p__Bacteroidetes.c__Bacteroidia.o__Bacteroidales.f__NA.g__NA.s__sp12401	SF	3.442	3.135	0.002
k__Bacteria.p__Proteobacteria.c__Alphaproteobacteria.o__Sphingomonadales.f__Sphingomonadaceae.g__Sphingomonas	SP	3.081	2.989	0.003
k__Bacteria.p__Proteobacteria.c__Gammaproteobacteria.o__Xanthomonadales.f__Xanthomonadaceae.g__Lysobacter.s__xinjiangensis	SF	3.050	2.885	0.004
k__Bacteria.p__Proteobacteria.c__Gammaproteobacteria.o__Xanthomonadales.f__Xanthomonadaceae.g__Lysobacter	SF	3.050	2.885	0.004
k__Bacteria.p__Firmicutes.c__Clostridia.o__Clostridiales.f__Ruminococcaceae.g__Eubacterium.s__sp34525	SF	2.669	2.641	0.004
k__Bacteria.p__Firmicutes.c__Clostridia.o__Clostridiales.f__Ruminococcaceae.g__Eubacterium	SF	2.698	2.607	0.004
k__Bacteria.p__Firmicutes.c__Bacilli.o__Lactobacillales.f__Leuconostocaceae	SF	3.407	3.107	0.006
k__Bacteria.p__Firmicutes.c__Clostridia.o__Clostridiales.f__Ruminococcaceae.g__NA.s__sp35155_sp35252	SF	3.009	2.841	0.007
k__Bacteria.p__Firmicutes.c__Clostridia.o__Clostridiales.f__Lachnospiraceae.g__NA.s__sp32945	SF	4.296	3.869	0.008
k__Bacteria.p__Firmicutes.c__Clostridia.o__Clostridiales.f__Ruminococcaceae.g__NA.s__sp35711	SF	2.605	2.878	0.008
k__Bacteria.p__Bacteroidetes.c__Bacteroidia.o__Bacteroidales.f__Prevotellaceae.g__NA.s__sp14143	SF	3.748	3.321	0.010
k__Bacteria.p__Firmicutes.c__Clostridia.o__Clostridiales.f__Ruminococcaceae.g__NA	SF	4.832	4.391	0.010
k__Bacteria.p__Firmicutes.c__Clostridia.o__Clostridiales.f__Christensenellaceae.g__NA.s__sp30299	SF	2.930	2.664	0.012
k__Bacteria.p__Firmicutes.c__Clostridia.o__Clostridiales.f__Lachnospiraceae.g__NA	SF	4.900	4.428	0.013
k__Bacteria.p__Firmicutes.c__Clostridia.o__Clostridiales.f__Ruminococcaceae	SF	4.922	4.413	0.013

k__Bacteria.p__Firmicutes.c__Clostridia.o__Clostridiales.f__Ruminococcaceae.g__NA.s__sp35374	SF	1.889	3.287	0.013
k__Bacteria.p__Bacteroidetes.c__Bacteroidia.o__Bacteroidales.f__Prevotellaceae.g__NA.s__sp13808	SF	2.778	2.807	0.013
k__Bacteria.p__Spirochaetae.c__Spirochaetes.o__Spirochaetales.f__Spirochaetaceae.g__NA.s__sp66714	SF	2.809	2.785	0.013
k__Bacteria.p__Firmicutes.c__Clostridia.o__Clostridiales.f__Ruminococcaceae.g__NA.s__sp35290	SF	2.217	2.771	0.013
k__Bacteria.p__Proteobacteria.c__Alphaproteobacteria.o__Rhodospirillales.f__Rhodospirillaceae	SF	2.802	2.667	0.013
k__Bacteria.p__Bacteroidetes.c__Flavobacteriia.o__Flavobacteriales.f__Flavobacteriaceae.g__Chryseobacterium.s__koreense	SF	2.627	2.744	0.013
k__Bacteria.p__Proteobacteria.c__Gammaproteobacteria.o__Pseudomonadales.f__Moraxellaceae.g__Acinetobacter.s__johnsonii	SF	3.315	3.082	0.014
k__Bacteria.p__Proteobacteria.c__Alphaproteobacteria.o__Sphingomonadales.f__Sphingomonadaceae	SP	3.114	2.792	0.014
k__Bacteria.p__Firmicutes.c__Clostridia.o__Clostridiales.f__Lachnospiraceae	SF	4.978	4.489	0.016
k__Bacteria.p__Bacteroidetes.c__Bacteroidia.o__Bacteroidales.f__NA	SF	4.446	4.012	0.016
k__Bacteria.p__Bacteroidetes.c__Bacteroidia.o__Bacteroidales.f__NA.g__NA	SF	4.421	3.983	0.016
k__Bacteria.p__Firmicutes.c__Bacilli.o__Lactobacillales.f__Lactobacillaceae.g__Lactobacillus	SF	3.956	3.396	0.016
k__Bacteria.p__Firmicutes.c__Bacilli.o__Lactobacillales.f__Streptococcaceae.g__Streptococcus.s__porcinus_uberis	SP	3.216	3.029	0.017
k__Bacteria.p__Firmicutes.c__Clostridia.o__Clostridiales.f__Ruminococcaceae.g__NA.s__sp33136	SF	3.701	3.381	0.017
k__Bacteria.p__Firmicutes.c__Clostridia.o__Clostridiales.f__Ruminococcaceae.g__NA.s__sp35645_sp35657	SF	2.901	2.749	0.017
k__Bacteria.p__Bacteroidetes.c__Bacteroidia.o__Bacteroidales.f__NA.g__NA.s__sp12449	SF	3.175	2.902	0.017
k__Bacteria.p__Bacteroidetes.c__Bacteroidia.o__Bacteroidales.f__Prevotellaceae.g__NA.s__sp14149	SF	3.823	3.353	0.018
k__Bacteria.p__Firmicutes.c__Bacilli.o__Lactobacillales.f__Lactobacillaceae	SF	3.956	3.385	0.019
k__Bacteria.p__Bacteroidetes.c__Bacteroidia.o__Bacteroidales.f__Prevotellaceae.g__NA	SF	4.782	4.328	0.019
k__Bacteria.p__Bacteroidetes.c__Flavobacteriia.o__Flavobacteriales.f__Flavobacteriaceae.g__Empedobacter	SF	3.541	3.186	0.019
k__Bacteria.p__Bacteroidetes.c__Bacteroidia.o__Bacteroidales.f__Rikenellaceae.g__NA.s__sp14588	SF	3.102	2.915	0.021

k__Bacteria.p__Bacteroidetes.c__Bacteroidia.o__Bacteroidales.f__Rikenellaceae.g__NA.s__sp14521	SF	3.008	2.820	0.021
k__Bacteria.p__Firmicutes.c__Bacilli.o__Lactobacillales.f__Lactobacillaceae.g__Lactobacillus.s__johnsonii	SF	2.834	2.655	0.021
k__Bacteria.p__Firmicutes.c__Bacilli.o__Lactobacillales.f__Lactobacillaceae.g__Lactobacillus.s__NA	SF	3.435	2.869	0.023
k__Bacteria.p__Bacteroidetes.c__Bacteroidia.o__Bacteroidales.f__NA.g__NA.s__sp14839	SF	3.811	3.461	0.023
k__Bacteria.p__Bacteroidetes.c__Flavobacteriia.o__Flavobacteriales.f__Flavobacteriaceae.g__Empedobacter.s__brevis_falsenii	SF	3.512	3.158	0.024
k__Bacteria.p__Firmicutes.c__Clostridia.o__Clostridiales.f__Lachnospiraceae.g__Blautia.s__sp32045	SF	2.749	2.748	0.027
k__Bacteria.p__Bacteroidetes.c__Bacteroidia.o__Bacteroidales.f__NA.g__NA.s__sp12490	SF	3.796	3.345	0.028
k__Bacteria.p__Tenericutes.c__Mollicutes	SF	3.496	3.173	0.028
k__Bacteria.p__Tenericutes	SF	3.496	3.173	0.028
k__Bacteria.p__Bacteroidetes.c__Bacteroidia.o__Bacteroidales.f__Prevotellaceae	SF	4.848	4.397	0.028
k__Bacteria.p__Firmicutes.c__Bacilli.o__Lactobacillales.f__Leuconostocaceae.g__Weissella.s__hellenica	SF	1.924	3.109	0.031
k__Bacteria.p__Firmicutes.c__Clostridia.o__Clostridiales.f__NA.g__NA.s__sp30551	SF	1.889	3.085	0.031
k__Bacteria.p__Bacteroidetes.c__Bacteroidia.o__Bacteroidales.f__Porphyromonadaceae.g__Parabacteroides.s__sp13254_sp13259_sp13270	SF	1.928	3.038	0.031
k__Bacteria.p__Firmicutes.c__Clostridia.o__Clostridiales.f__Ruminococcaceae.g__NA.s__sp35320	SF	1.908	2.998	0.031
k__Bacteria.p__Lentisphaerae.c__NA.o__NA.f__NA.g__NA.s__sp38872	SF	2.046	2.992	0.031
k__Bacteria.p__Firmicutes.c__Clostridia.o__Clostridiales.f__Lachnospiraceae.g__Lachnoclostridium.s__sp32440	SF	1.950	2.981	0.031
k__Bacteria.p__Actinobacteria.c__Actinobacteria.o__Propionibacteriales.f__Nocardioidaceae.g__NA	SF	2.543	2.977	0.031
k__Bacteria.p__Actinobacteria.c__Actinobacteria.o__Propionibacteriales.f__Nocardioidaceae.g__NA.s__sp7702	SF	2.543	2.966	0.031
k__Bacteria.p__Firmicutes.c__Clostridia.o__Clostridiales.f__Ruminococcaceae.g__NA.s__sp35639	SF	2.289	2.940	0.031
k__Bacteria.p__Actinobacteria.c__Actinobacteria.o__Frankiales	SF	2.227	2.772	0.031
k__Bacteria.p__Firmicutes.c__Clostridia.o__Clostridiales.f__Ruminococcaceae.g__NA.s__sp35683	SF	2.166	2.759	0.031

k__Bacteria.p__Firmicutes.c__Bacilli.o__Lactobacillales.f__Leuconostocaceae.g__Weissella.s__cibaria_confusa	SF	2.918	2.727	0.031
k__Bacteria.p__Firmicutes.c__Clostridia.o__Clostridiales.f__NA.g__NA.s__sp31099	SF	2.328	2.710	0.031
k__Bacteria.p__Firmicutes.c__Clostridia.o__Clostridiales.f__Family_XIII.g__Anaerovorax.s__sp31505_sp31510	SF	2.352	2.690	0.031
k__Bacteria.p__Firmicutes.c__Clostridia.o__Clostridiales.f__Ruminococcaceae.g__NA.s__sp35645	SF	2.664	2.687	0.031
k__Bacteria.p__Tenericutes.c__Mollicutes.o__NA.f__NA.g__NA.s__sp67804	SF	2.230	2.666	0.031
k__Bacteria.p__Firmicutes.c__Clostridia.o__Clostridiales.f__Ruminococcaceae.g__NA.s__sp35360	SF	2.510	2.637	0.031
k__Bacteria.p__Firmicutes.c__Bacilli.o__Lactobacillales.f__Lactobacillaceae.g__Lactobacillus.s__reuteri	SF	3.227	2.898	0.032
k__Bacteria.p__Firmicutes.c__Clostridia.o__Clostridiales.f__Lachnospiraceae.g__NA.s__sp32661	SF	3.613	3.213	0.033
k__Bacteria.p__Bacteroidetes.c__Bacteroidia.o__Bacteroidales.f__Prevotellaceae.g__NA.s__sp14133	SF	3.631	3.382	0.033
k__Bacteria.p__Firmicutes.c__Clostridia.o__Clostridiales.f__Ruminococcaceae.g__NA.s__sp35637	SF	2.684	3.025	0.033
k__Bacteria.p__Firmicutes.c__Clostridia.o__Clostridiales.f__Lachnospiraceae.g__NA.s__sp32463	SF	4.093	3.603	0.034
k__Bacteria.p__Firmicutes.c__Clostridia	SF	5.434	4.706	0.034
k__Bacteria.p__Firmicutes.c__Clostridia.o__Clostridiales	SF	5.434	4.706	0.034
k__Bacteria.p__Proteobacteria.c__Alphaproteobacteria.o__Sphingomonadales.f__Sphingomonadaceae.g__Sphingomonas.s__adhaesiva_ginsenosidimutans	SP	2.861	3.152	0.036
k__Bacteria.p__Tenericutes.c__Mollicutes.o__NA.f__NA.g__NA.s__sp67801	SF	2.279	2.816	0.036
k__Bacteria.p__Firmicutes.c__Clostridia.o__Clostridiales.f__Peptostreptococcaceae.g__Terrisporobacter	SF	3.497	2.902	0.037
k__Bacteria.p__Firmicutes.c__Clostridia.o__Clostridiales.f__Ruminococcaceae.g__NA.s__sp35117	SF	3.460	3.062	0.037
k__Bacteria.p__Firmicutes.c__Clostridia.o__Clostridiales.f__Lachnospiraceae.g__NA.s__sp32624_sp32794	SF	2.921	2.751	0.039
k__Bacteria.p__Tenericutes.c__Mollicutes.o__NA.f__NA.g__NA	SF	3.163	2.911	0.041
k__Bacteria.p__Tenericutes.c__Mollicutes.o__NA	SF	3.163	2.911	0.041
k__Bacteria.p__Tenericutes.c__Mollicutes.o__NA.f__NA	SF	3.163	2.911	0.041
k__Bacteria.p__Firmicutes.c__Bacilli.o__Lactobacillales.f__Streptococcaceae.g__Streptococcus.s__NA	SF	4.354	3.710	0.041

k__Bacteria.p__Firmicutes.c__Clostridia.o__Clostridiales.f__Family_XI.g__Anaerococcus.s__sp31221	SF	2.815	2.858	0.041
k__Bacteria.p__Tenericutes.c__Mollicutes.o__Mycoplasmatales.f__Mycoplasmataceae	SF	3.112	3.023	0.045
k__Bacteria.p__Tenericutes.c__Mollicutes.o__Mycoplasmatales	SF	3.112	3.021	0.045
k__Bacteria.p__Firmicutes.c__Clostridia.o__Clostridiales.f__Ruminococcaceae.g__NA.s__sp35612	SF	3.108	2.951	0.045
k__Bacteria.p__Firmicutes.c__Clostridia.o__Clostridiales.f__Family_XIII.g__Anaerovorax	SF	2.442	2.904	0.045
k__Bacteria.p__Firmicutes.c__Clostridia.o__Clostridiales.f__Ruminococcaceae.g__Fastidiosipila.s__sp34560	SP	3.083	2.991	0.046
k__Bacteria.p__Firmicutes.c__Bacilli.o__Lactobacillales.f__Streptococcaceae.g__Streptococcus.s__alactolyticus	SF	3.400	2.894	0.046
k__Bacteria.p__Firmicutes.c__Clostridia.o__Clostridiales.f__Ruminococcaceae.g__NA.s__sp35046	SF	3.461	3.114	0.048
k__Bacteria.p__Firmicutes.c__Clostridia.o__Clostridiales.f__Lachnospiraceae.g__NA.s__sp33775	SF	3.023	2.906	0.048
k__Bacteria.p__Firmicutes.c__Clostridia.o__Clostridiales.f__NA.g__NA	SF	3.813	3.453	0.048
k__Bacteria.p__Bacteroidetes.c__Bacteroidia.o__Bacteroidales.f__Prevotellaceae.g__NA.s__sp14130	SF	3.740	3.346	0.048
k__Bacteria.p__Firmicutes.c__Clostridia.o__Clostridiales.f__NA	SF	3.839	3.445	0.049
k__Bacteria.p__Actinobacteria.c__Actinobacteria.o__Corynebacteriales.f__Nocardiaceae.g__Rhodococcus	SP	4.391	3.977	0.049
k__Bacteria.p__Actinobacteria.c__Actinobacteria.o__Corynebacteriales.f__Nocardiaceae	SP	4.391	3.977	0.049
k__Bacteria.p__Actinobacteria.c__Actinobacteria.o__Actinomycetales.f__Actinomycetaceae.g__NA.s__sp4821	SF	2.756	2.912	0.050
k__Bacteria.p__Firmicutes.c__Clostridia.o__Clostridiales.f__Ruminococcaceae.g__NA.s__sp35504	SF	2.952	2.702	0.050
